# Artificial intelligence for autism spectrum disorder: advances in diagnosis, behavior analysis and educational support

**DOI:** 10.3389/fnins.2026.1832743

**Published:** 2026-06-12

**Authors:** José Jesús Sánchez Amate, Antonio Luque de la Rosa

**Affiliations:** Department of Education, University of Almería, Almería, Spain

**Keywords:** artificial intelligence, autism spectrum disorder, early detection, educational technology, machine learning

## Abstract

**Introduction:**

Artificial intelligence has become an increasingly relevant field of research in the study of Autism Spectrum Disorder (ASD), offering novel technological approaches for the analysis, detection, and support of individuals on the autism spectrum. The aim of this study was to systematically review recent scientific literature examining the application of artificial intelligence in ASD.

**Methods:**

The review was conducted following the PRISMA 2020 guidelines. Searches were performed in PubMed, Scopus, Dialnet, and Google Scholar, including studies published between 2019 and 2025. After applying predefined inclusion and exclusion criteria, 18 empirical studies were included in the final analysis. Methodological quality and risk of bias were assessed using Joanna Briggs Institute critical appraisal tools adapted to the methodological design of each study.

**Results:**

Current research focuses primarily on four areas: early detection and diagnostic support, automated analysis of behavioral and social patterns, AI-based educational technologies, and communication support systems. Although the reviewed studies demonstrate promising advances in machine learning, computer vision, and natural language processing, important methodological limitations remain, particularly regarding external validation, dataset representativeness, and heterogeneity of performance indicators.

**Discussion:**

Overall, artificial intelligence shows considerable potential for supporting diagnosis, education, and communication in ASD; however, greater methodological robustness, transparency, and ethical safeguards remain necessary before broader implementation in real clinical and educational settings.

## 1 Introduction

Autism Spectrum Disorder (ASD) is a neurodevelopmental condition characterized by persistent differences in social communication, interpersonal interaction, and the presence of restricted or repetitive patterns of behavior (American Psychiatric Association, 2013). These characteristics manifest with varying levels of intensity and generate highly heterogeneous cognitive and behavioral profiles within the autism spectrum, which implies diverse educational and support needs (Lord et al., 2020).

Over recent decades, scientific interest in understanding the cognitive, social, and educational processes associated with autism has increased considerably. At the same time, the development of digital technologies has created new opportunities to design intervention and support tools that can respond more precisely to the individual characteristics of people with ASD (Parsons and Cobb, 2011). In this context, emerging technologies have begun to play an increasingly significant role in both educational and clinical settings.

Among these technological innovations, Artificial Intelligence (AI) has emerged as one of the fields with the greatest potential for application in autism research. AI-based systems enable the analysis of large volumes of data and the identification of complex behavioral patterns, facilitating the development of predictive models and adaptive support tools (Russell and Norvig, 2021). Within autism research, these technologies have been applied in several areas, including early detection of the disorder, automated analysis of social behavior, and the development of personalized educational tools (Thabtah, 2019).

Several studies have explored the use of machine learning algorithms to identify early indicators of autism using behavioral, linguistic, or biometric data. In some cases, these models have demonstrated the ability to detect patterns associated with ASD through the analysis of facial expressions, speech characteristics, or visual attention patterns (Duda et al., 2016). Such technological advances have generated growing interest in the development of tools capable of supporting early screening and assisting clinical decision-making processes, although these systems should not be understood as substitutes for formal clinical diagnosis conducted by healthcare professionals.

Similarly, the application of artificial intelligence in educational contexts has enabled the development of adaptive learning environments capable of adjusting pedagogical activities to the cognitive and behavioral characteristics of each student. These intelligent educational platforms can provide immediate feedback, adapt the level of task difficulty, and promote greater participation of students with ASD in inclusive educational settings (Chen et al., 2016). In this way, artificial intelligence is emerging as a promising tool for enhancing teaching and learning processes for this population.

However, the integration of artificial intelligence in the field of autism also raises important challenges. These include issues related to data ethics, potential biases in algorithms, and inequalities in access to technological resources. Several authors have emphasized the need to develop ethical and regulatory frameworks to ensure the responsible use of artificial intelligence in social and educational contexts (Floridi and Cowls, 2019).

From a neurodiversity perspective, autism should not be understood solely as a clinical disorder but also as a form of neurological diversity that forms part of human variability. This perspective promotes a broader understanding of autism, emphasizing the recognition of cognitive differences and the development of more inclusive social and educational environments (Singer, 2017). In this sense, AI-based technologies should be designed not only to address specific challenges but also to enhance cognitive strengths and support the social participation of autistic individuals.

Despite the growing scientific interest in this field, the literature on artificial intelligence and autism remains heterogeneous and dispersed. Existing research addresses multiple technological, methodological, and educational approaches, making it difficult to obtain an integrated overview of the current state of knowledge. For this reason, a systematic review of the literature is necessary in order to jointly analyze the available evidence on the use of artificial intelligence in the field of Autism Spectrum Disorder.

Recent systematic reviews and meta-analyses have begun to examine specific applications of artificial intelligence in Autism Spectrum Disorder (ASD). Recent evidence has explored the role of AI in supporting autism diagnosis and behavioral monitoring through machine learning–based predictive systems, social behavior tracking, and automated video analysis for early detection (Sun et al., 2025; Aldakhil and Alasim, 2025; Jin et al., 2024). Although these studies provide valuable insights into specific technological domains, much of the recent literature remains concentrated on relatively narrow areas, particularly diagnostic prediction and behavioral classification. Consequently, there remains a need for broader integrative reviews capable of simultaneously examining diagnostic, behavioral, educational, and communication-related applications of artificial intelligence in ASD. In this regard, the present review aims to provide a more comprehensive understanding of how AI is currently being applied across multiple dimensions of autism support and intervention.

Within this context, the aim of this systematic review is to examine the main applications of artificial intelligence in Autism Spectrum Disorder and to identify the principal research trends and challenges associated with its implementation in educational and clinical contexts.

## 2 Materials and methods

### 2.1 Research strategy

This study was conducted as a systematic review of the literature in accordance with the Preferred Reporting Items for Systematic Reviews and Meta-Analyses (PRISMA) guidelines, which are widely used to ensure transparency and methodological rigor in systematic reviews of scientific research (Page et al., 2021). As the study was based exclusively on the analysis of previously published studies, approval from an ethics committee or Institutional Review Board was not required.

To identify relevant studies on the application of artificial intelligence in the field of Autism Spectrum Disorder (ASD), a comprehensive literature search was performed using four major international databases: PubMed, Dialnet, Scopus, and Google Scholar. These databases were selected due to their broad coverage of research in the fields of medicine, psychology, education, and health-related technologies.

The search covered studies published between January 2019 and May 2025, with the aim of identifying recent research related to the development and application of artificial intelligence–based technologies in the field of autism.

To identify relevant studies, a structured search strategy was developed and adapted to the indexing characteristics of each database. Boolean operators (“AND,” “OR”) were used to combine descriptors related to Autism Spectrum Disorder and Artificial Intelligence (AI), with the aim of maximizing the identification of relevant studies. The principal search equation applied in PubMed and Scopus was: (“Autism Spectrum Disorder” OR ASD OR autism) AND (“Artificial Intelligence” OR AI OR “Machine Learning” OR “Deep Learning” OR “Computer Vision” OR “Natural Language Processing”) AND (diagnosis OR detection OR education OR intervention OR behavior OR communication). For Dialnet and Google Scholar, simplified combinations of descriptors were used due to differences in indexing systems and search functionalities. These included terms such as “autism and artificial intelligence,” “autism and machine learning,” “artificial intelligence and autism diagnosis,” “AI-based autism education,” “machine learning and autism detection,” “artificial intelligence and autism behavior analysis,” and “AI and autism communication support.” Searches were conducted independently in each database, and filters were applied to restrict results to studies published in English between 2019 and 2025 with full-text availability.

The complete search strategies used for each database, including Boolean operators, filters, search dates, and retrieved records, are available in Supplementary Table 1 to ensure full reproducibility and transparency of the review process. Compliance with PRISMA 2020 reporting recommendations was further documented through the completed PRISMA 2020 Checklist, provided in Supplementary Table 1.

This systematic review was not prospectively registered in PROSPERO or another international review registry. Given the interdisciplinary and exploratory nature of the topic, the review protocol was developed by the authors following PRISMA 2020 methodological recommendations. The absence of prospective registration is acknowledged as a methodological limitation.”

The initial search identified 298 potentially relevant records. After removing duplicates and clearly irrelevant records, titles and abstracts were screened to determine their eligibility for inclusion in the review.

Two researchers independently reviewed the studies identified through the literature search. Duplicate records were removed, and relevant information from each article was recorded in a spreadsheet created using Microsoft Excel (Microsoft Corporation, Redmond, WA, United States). The results obtained by both reviewers were subsequently compared, and any discrepancies in the study selection process were discussed until consensus was reached. Only studies on which both reviewers reached agreement were included in the final analysis.

**Figure 1:**
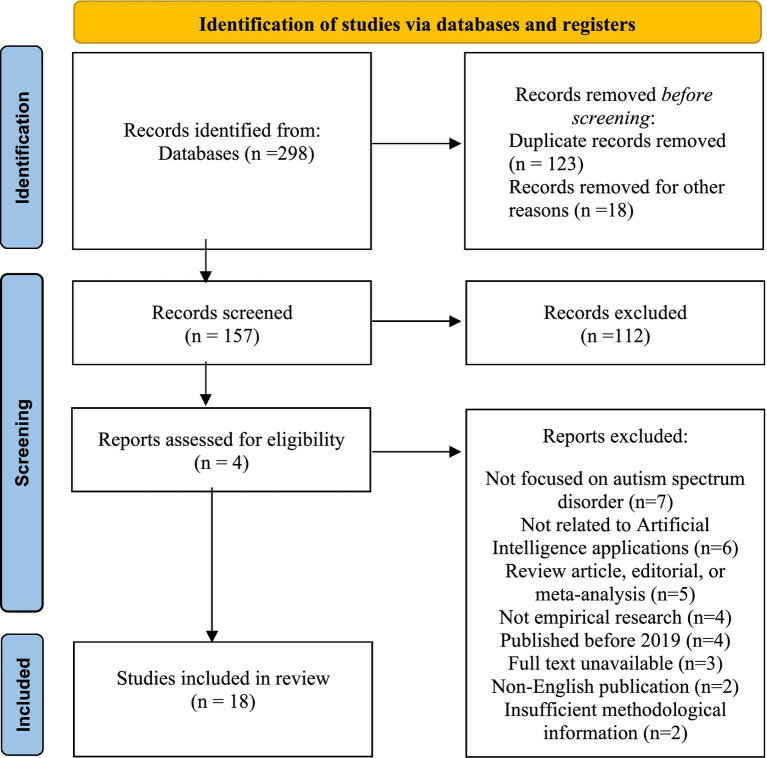
Flowchart of study selection.

The complete process of study identification, screening, and eligibility is presented in Figure 1, following the structure of the PRISMA flow diagram.

### 2.2 Study selection criteria

Inclusion and exclusion criteria were established to ensure the quality and relevance of the studies included in the review.

The inclusion criteria were as follows:

- Empirical studies published in peer-reviewed scientific journals
- Articles written in English
- Studies focusing on the application of artificial intelligence in the field of Autism Spectrum Disorder (ASD)
- Studies published between 2019 and 2025
- Availability of full-text access

Studies with different methodological designs were included, such as observational studies, experimental studies, longitudinal research, and technological validation studies examining the use of artificial intelligence for autism detection, behavioral analysis, educational intervention, or communication support.

Several exclusion criteria were also established. Articles focusing exclusively on pharmacological treatments for autism or clinical interventions unrelated to digital technologies were excluded. Publications written in languages other than English, single case studies, case series with very small samples, narrative reviews, previous systematic reviews, and meta-analyses were also excluded.

In addition, studies published before 2019 were excluded in order to focus the analysis on the most recent developments in artificial intelligence applied to autism.

After applying the selection criteria, the eligible articles were assessed through full-text review to determine their relevance to the objectives of the study. During this stage, relevant variables were extracted from each study, including author, year of publication, sample characteristics, methodology, type of artificial intelligence technology used, and the main findings.

A total of 18 studies met the established criteria and were included in the final systematic review.

### 2.3 Quality assessment and risk of bias

To assess the methodological quality and potential risk of bias of the included studies, a structured critical appraisal was conducted using the Joanna Briggs Institute (JBI) Critical Appraisal Checklists. Given the methodological heterogeneity of the studies included in this systematic review, the checklist most appropriate to each study design was selected. Diagnostic accuracy studies were assessed using the JBI Critical Appraisal Checklist for Diagnostic Test Accuracy Studies, intervention-based studies were assessed using the JBI Checklist for Randomized Controlled Trials or Quasi-Experimental Studies, and observational or dataset-based studies were assessed using the JBI Checklist for Analytical Cross-Sectional Studies. Two reviewers independently assessed the methodological quality of the included studies, and disagreements were resolved through discussion until consensus was reached. The results of the quality assessment are presented in Supplementary Table 2.

### 2.4 Statistical analysis

Since the main objective of this study was to synthesize the available scientific evidence on the application of artificial intelligence in the field of Autism Spectrum Disorder (ASD), the data extracted from the selected studies were analyzed using a descriptive and comparative approach.

The included studies were grouped into different thematic categories according to their main area of application. These categories included early detection and diagnostic support for autism, automated analysis of social behavior, the development of AI-based educational tools, and communication support technologies.

A comparative analysis of the findings reported in the selected studies was then conducted in order to identify common patterns, research trends, and potential methodological differences among the studies analyzed. Whenever available, performance indicators reported in the selected studies, including accuracy, sensitivity, specificity, and predictive performance measures, were considered to complement the qualitative synthesis of findings.

Due to the heterogeneity of the methodological designs, the variables analyzed, and the indicators used to evaluate outcomes, it was not possible to conduct a quantitative meta-analysis. Therefore, the results are presented in Table 1 through a narrative synthesis that summarizes the main evidence identified in the scientific literature.

**Table 1:** Complete search strategy across databases used in the systematic review.

Database	Date of search	Complete search strategy	Filters applied	Initial records retrieved (n)
PubMed	May 2025	(“Autism Spectrum Disorder” OR ASD OR autism) AND (“Artificial Intelligence” OR AI OR “Machine Learning” OR “Deep Learning” OR “Computer Vision” OR “Natural Language Processing”) AND (diagnosis OR detection OR education OR intervention OR behavior OR communication)	2019–2025; full text	86
Scopus	May 2025	TITLE-ABS-KEY((“Autism Spectrum Disorder” OR ASD OR autism) AND (“Artificial Intelligence” OR AI OR “Machine Learning” OR “Deep Learning” OR “Computer Vision” OR “Natural Language Processing”) AND (diagnosis OR detection OR education OR intervention OR behavior OR communication))	2019–2025; articles; full text	112
Dialnet	May 2025	“autism and artificial intelligence” OR “autism and machine learning” OR “artificial intelligence and autism diagnosis” OR “AI-based autism education” OR “machine learning and autism detection” OR “artificial intelligence and autism behavior analysis” OR “AI and autism communication support”	2019–2025; scientific articles; full text	28
Google scholar	May 2025	“autism and artificial intelligence”; “autism and machine learning”; “artificial intelligence and autism diagnosis”; “AI-based autism education”; “machine learning and autism detection”; “artificial intelligence and autism behavior analysis”; “AI and autism communication support”	2019–2025; English; relevance-based screening; full text when available	72
Total	298			

## 3 Results

### 3.1 Identification of the selected publications

The methodological quality of the included studies ranged from moderate to high. Overall, studies focusing on diagnostic accuracy and machine learning-based classification showed generally adequate methodological quality, although several limitations were identified, particularly related to sample representativeness, limited external validation, dataset imbalance, and the risk of overfitting. Educational and communication-support studies presented moderate methodological quality, mainly due to small sample sizes, limited longitudinal follow-up, and restricted validation in real-world educational contexts. Detailed results of the quality assessment and risk of bias appraisal are presented in Supplementary Table 2.

Within the category of early detection and diagnostic support, machine learning algorithms were identified as the most prominent line of research among the selected studies. Abbas et al. (2019) report that artificial intelligence–based models are capable of identifying behavioral patterns associated with Autism Spectrum Disorder with a high level of accuracy in automated classification contexts. Similarly, Thabtah (2019) highlights that machine learning algorithms improve screening processes by systematically analyzing behavioral variables related to autism. In the same vein, Tariq et al. (2019) demonstrate that digital platforms based on artificial intelligence can facilitate the evaluation of behavioral indicators associated with ASD through the automated analysis of behavioral data.

Likewise, Thabtah and Peebles (2020) developed predictive models capable of classifying characteristics associated with autism with high levels of accuracy. In a similar direction, Alharbi et al. (2022) report that machine learning models applied to behavioral datasets may support screening and diagnostic decision-making processes through the identification of behavioral patterns associated with ASD. More recent studies reinforce these findings. Rasul et al. (2024) emphasize that machine learning models can partially automate diagnostic processes through the identification of relevant behavioral patterns. Furthermore, Jiang et al. (2025) propose an explainable artificial intelligence framework that improves the interpretability of diagnostic models, enabling a clearer understanding of how algorithms make decisions in clinical contexts.

Another relevant line of research identified in the literature focuses on the early detection of autism through the automated analysis of behavioral and audiovisual data. Voss et al. (2019) indicate that the analysis of behavioral markers using digital technologies can contribute to the early identification of ASD. Similarly, Tariq et al. (2022) demonstrate that mobile tools based on artificial intelligence can detect early indicators of autism through the analysis of observable behaviors. More recently, Kim et al. (2025) show that deep learning models applied to video analysis can improve the accuracy of early autism detection by identifying specific behavioral patterns associated with the disorder.

Another important research line corresponds to the automated analysis of behavioral and social patterns using computer vision techniques. Washington et al. (2020) demonstrate that artificial intelligence–based systems can analyze differences in social interaction patterns between children with and without autism. In the same line, Levy et al. (2017) report that automated analysis of facial expressions allows the identification of emotional patterns characteristic of individuals with ASD. Likewise, Washington et al. (2021) show that computer vision algorithms can analyze variables related to visual attention and social behavior during interaction.

In the educational domain, the reviewed studies indicate that artificial intelligence–based technologies can contribute to the development of more adaptive learning environments for students with autism. Chen et al. (2021) report that AI-based educational tools can improve the development of social and emotional skills through interactive learning environments adapted to the characteristics of students.

Finally, the communication support category is represented by studies focusing on natural language processing. Bone et al. (2020) identify significant differences in linguistic patterns associated with autism through artificial intelligence techniques, suggesting that these systems may contribute to the development of more precise communication support tools tailored to the needs of individuals with ASD (Table 2).

**Table 2:** Quality assessment and risk of bias of included studies.

Study	Main design/focus	JBI tool used	Score	Quality level	Main risk of bias identified
Abbas et al. (2019)	Machine learning/ASD detection	JBI analytical cross-sectional checklist	6/8	Moderate	Limited external validation and possible dataset-specific bias
Thabtah (2019)	Behavioral machine learning study	JBI analytical cross-sectional checklist	6/8	Moderate	Heterogeneity of behavioral variables and limited generalizability
Tariq et al. (2019)	Home-video ML screening	JBI diagnostic accuracy checklist	7/9	High	Potential selection bias and reliance on video quality
Voss et al. (2019)	Wearable digital intervention	JBI randomized controlled trial checklist	10/13	Moderate–High	Limited follow-up and implementation constraints
Thabtah and Peebles (2020)	Rule-based ML autism detection	JBI analytical cross-sectional checklist	6/8	Moderate	Limited external validation and risk of overfitting
Bone et al. (2020)	ML/NLP screening and diagnosis	JBI diagnostic accuracy checklist	7/9	High	Variability in linguistic and behavioral measures
Washington et al. (2020)	Computer vision/behavioral feature extraction	JBI diagnostic accuracy checklist	7/9	High	Limited ecological validation and sample representativeness
Chen et al. (2021)	AI-supported educational intervention	JBI quasi-experimental checklist	7/9	Moderate–High	Limited sample size and contextual specificity
Levy et al. (2017)	Automated facial/emotional analysis	JBI analytical cross-sectional checklist	6/8	Moderate	Limited diversity of samples and possible measurement bias
Washington et al. (2021)	Data-driven diagnostics/AI	JBI diagnostic accuracy checklist	7/9	High	Generalizability across clinical populations remains limited
Alharbi et al. (2022)	ML detection using behavioral datasets	JBI analytical cross-sectional checklist	6/8	Moderate	Dataset imbalance and limited independent validation
Tariq et al. (2022)	ML analysis of home videos	JBI diagnostic accuracy checklist	7/9	High	Dependence on caregiver-recorded videos and sampling bias
Hu and Han (2022)	Intelligent tutoring systems	JBI quasi-experimental checklist	6/9	Moderate	Limited real-world educational validation
Wang et al. (2023)	AI-supported speech generation	JBI quasi-experimental checklist	6/9	Moderate	Small sample and limited longitudinal follow-up
Lan et al. (2024)	AI in ASD education	JBI analytical cross-sectional checklist	6/8	Moderate	Heterogeneous educational contexts and limited outcome standardization
Rasul et al. (2024)	ML approaches for early diagnosis	JBI diagnostic accuracy checklist	7/9	High	Need for broader external validation
Jiang et al. (2025)	Explainable AI/multimodal diagnosis	JBI diagnostic accuracy checklist	8/9	High	Emerging evidence with limited independent replication
Kim et al. (2025)	Deep learning/home-video ASD identification	JBI diagnostic accuracy checklist	8/9	High	Potential bias linked to video selection and dataset composition

Overall, the selected studies show that artificial intelligence is primarily applied in four main domains: early detection and diagnostic support, behavioral and social analysis, educational intervention, and the development of communication support tools. Although several studies reported promising levels of predictive performance, including accuracy and classification effectiveness, methodological limitations related to sample size, heterogeneity of research designs, and variability in reported performance indicators limited direct comparison across studies and prevented quantitative synthesis.

Table 3 summarizes the main findings derived from the systematic review on the application of artificial intelligence in Autism Spectrum Disorder. The table organizes the most relevant categories identified in the literature together with the studies supporting each of them, providing an integrated overview of the main research trends in this field.

**Table 3:** Description of the included studies.

Author/Year	Objective	Participants	Methodology	Area of application	Results
Abbas et al. (2019)	To develop an AI-based model for identifying behavioral patterns associated with ASD	97	Mixed-methods methodology	Diagnostic	The model enabled the classification of behaviors associated with ASD with high accuracy
Thabtah (2019)	To evaluate machine learning algorithms applied to autism diagnosis	200	Quantitative methodology	Diagnostic	The algorithms identified behavioral features relevant to the diagnosis of ASD
Voss et al. (2019)	To analyze early behavioral markers using artificial intelligence	85	Mixed-methods methodology	Early detection	The system enabled the identification of early indicators of ASD
Tariq et al. (2019)	To design an AI-based digital platform for behavioral assessment	162	Mixed-methods methodology	Diagnostic	The tool demonstrated high sensitivity in detecting characteristics of ASD
Voss et al. (2019)	To analyze the effect of an artificial intelligence-based digital intervention aimed at improving socialization in children with ASD	71	Mixed-methods methodology	Communication support	Significant improvement in social interaction and communication skills through the use of intelligent digital devices
Washington et al. (2020)	To use computer vision to analyze patterns of social interaction	95	Mixed-methods methodology	Behavioral analysis	The system identified differences in social interaction between children with ASD
Thabtah and Peebles (2020)	To compare different predictive models for the diagnosis of ASD	110	Quantitative methodology	Diagnostic	The models demonstrated high accuracy in data classification
Bone et al. (2020)	To analyze linguistic patterns in individuals with ASD using artificial intelligence	120	Mixed-methods methodology	Communication	Identification of relevant linguistic differences
Levy et al. (2017)	To analyze facial expressions using artificial intelligence	65	Mixed-methods methodology	Behavioral analysis	Automatic identification of emotional patterns
Washington et al. (2021)	To analyze social behaviors using computer vision algorithms	70	Qualitative methodology	Social interaction	The system detected differences in visual attention patterns
Chen et al. (2021)	To evaluate an AI-based educational tool designed to improve social skills	34	Quantitative methodology	Educational intervention	Improvement in emotional expression and social skills
Alharbi et al. (2022)	To develop a machine learning–based predictive model for diagnosis	58	Mixed-methods methodology	Diagnostic	Automated classification of behaviors associated with ASD
Tariq et al. (2022)	To evaluate an AI-based mobile tool for early detection	312	Quantitative methodology	Early detection	High accuracy in the identification of early indicators
Hu and Han (2022)	To develop an artificial intelligence–based intelligent tutoring system to support the learning of students with ASD	45	Mixed-methods methodology	Educational intervention	The adaptive system enabled the personalization of educational activities and improved the acquisition of social skills
Wang et al. (2023)	To evaluate an artificial intelligence–assisted speech generation system to improve communication in children with ASD	38	Mixed-methods methodology	Communication support	Improvement in verbal communication and social interaction through the use of AI-assisted language generation tools
Rasul et al. (2024)	To develop machine learning models for automatic diagnosis	150	Quantitative methodology	Diagnostic	Improvement in diagnostic automation
Kim et al. (2025)	A video-based early detection system using deep learning	120	Mixed-methods methodology	Early detection	Improved diagnostic accuracy through the use of artificial intelligence
Jiang et al. (2025)	An explainable artificial intelligence framework for autism diagnosis	100	Quantitative methodology	Diagnostic	Improved interpretability of artificial intelligence models

## 4 Discussion

The findings of this systematic review highlight the growing expansion of artificial intelligence (AI) applications in the field of Autism Spectrum Disorder (ASD), reflecting a progressive transition from exploratory technological experimentation toward increasingly structured approaches with potential clinical and educational applicability. Overall, the studies analyzed suggest that artificial intelligence is being integrated into four major domains: early detection and diagnostic support, automated behavioral and social analysis, educational intervention, and communication support. However, rather than representing a homogeneous field of development, the literature reveals substantial methodological diversity, technological variability, and differing levels of empirical validation. Consequently, the interpretation of the available evidence requires a critical and integrative perspective capable of examining not only the technological promise of AI systems but also their methodological robustness, practical applicability, limitations, and ethical implications (Table 4).

**Table 4:** Maps of evidences.

Category	Reference/year	Type of study	Key findings
Early detection and diagnostic support	Abbas et al. (2019)	Observational	Automated identification of behavioral patterns associated with ASD using machine learning algorithms
	Thabtah (2019)	Experimental	Machine learning models improve autism screening processes
	Alharbi et al. (2022)	Observational	Improved diagnostic accuracy through the use of machine learning models
	Tariq et al. (2019)	Experimental	An artificial intelligence–based digital platform for the behavioral assessment of ASD
	Thabtah and Peebles (2020)	Experimental	Predictive models with high accuracy in the classification of autism-related data
	Rasul et al. (2024)	Experimental	Improved automation of autism diagnosis through machine learning models
	Jiang et al. (2025)	Experimental	Development of an explainable artificial intelligence framework to improve the interpretability of diagnostic models for ASD
	Voss et al. (2019)	Observational	Identification of early behavioral markers associated with ASD
	Tariq et al. (2022)	Experimental	An artificial intelligence–based mobile application with high sensitivity for early detection
	Kim et al. (2025)	Experimental	A video-based early detection system using deep learning that improves diagnostic accuracy
Behavioral and social analysis	Washington et al. (2020)	Experimental	Identification of social patterns using computer vision
	Levy et al. (2017)	Observational	Automated analysis of facial expressions to examine emotional processing
	Washington et al. (2021)	Observational	Automatic detection of differences in social interaction and visual attention
Educational intervention	Chen et al. (2021)	Experimental	Artificial intelligence–based educational tools promote the development of social skills
	Hu and Han (2022)	Experimental	An artificial intelligence–based intelligent tutoring system that adapts educational activities to the individual needs of students with ASD, facilitating the acquisition of social skills
Communication support	Bone et al. (2020)	Experimental	Identification of linguistic patterns associated with ASD using artificial intelligence
	Voss et al. (2019)	Experimental	An artificial intelligence–based digital system that analyzes patterns of social interaction and communication using sensors and wearable devices
	Wang et al. (2023)	Experimental	An artificial intelligence–assisted speech generation system that improves verbal communication and social interaction in children with ASD

One of the principal findings emerging from the present review concerns the heterogeneity of artificial intelligence approaches applied to ASD. The reviewed literature includes classical machine learning models, deep learning architectures, computer vision systems, natural language processing tools, and multimodal approaches integrating several sources of behavioral data. Importantly, these technological approaches differ considerably in their methodological requirements, interpretability, predictive performance, and potential applicability within clinical and educational contexts.

Studies based on classical machine learning approaches, including those reported by Abbas et al. (2019), Thabtah (2019), Thabtah and Peebles (2020), and Alharbi et al. (2022), primarily relied on structured behavioral datasets to classify characteristics associated with ASD. These models often used behavioral questionnaires or predefined variables to identify patterns related to autism symptomatology. One advantage of traditional machine learning approaches lies in their relative interpretability, allowing researchers and professionals to better understand the variables contributing to prediction outcomes. In clinical contexts, interpretability represents an important advantage because healthcare professionals require transparency in diagnostic support systems, particularly when decisions affect access to specialized intervention or educational accommodations. However, despite their promising predictive capabilities, many of these studies relied on relatively small datasets and limited external validation procedures, which raises concerns regarding generalizability across diverse populations and contexts.

In contrast, deep learning approaches demonstrated increasing relevance, particularly in studies involving video analysis and behavioral pattern recognition. Kim et al. (2025), for example, illustrate how deep learning systems can analyze home videos to identify behavioral indicators associated with autism with high levels of predictive performance. Similarly, computer vision–based approaches examined by Washington et al. (2020, 2021) focused on the automated extraction of behavioral markers related to social interaction, visual attention, and eye contact. Compared with traditional machine learning, deep learning systems may achieve superior predictive accuracy due to their capacity to process large volumes of complex and unstructured data, including audiovisual information. Nevertheless, these advantages are accompanied by important methodological and practical challenges. Deep learning models often require extensive training datasets and substantial computational resources, while simultaneously presenting lower levels of interpretability. In clinical and educational environments, the “black box” nature of some AI systems may limit trust among practitioners and families, particularly when decision-making processes remain insufficiently transparent.

The increasing incorporation of explainable artificial intelligence frameworks represents an important development in this regard. Jiang et al. (2025) demonstrate how explainable AI approaches may improve transparency by clarifying how predictive decisions are generated through multimodal behavioral information. From a practical perspective, explainability may be especially important in ASD contexts because diagnostic and educational decisions frequently involve multidisciplinary teams requiring interpretable and accountable evidence. Consequently, explainable AI may represent an important pathway toward the responsible integration of machine learning systems into professional practice.

Natural language processing (NLP) approaches also represent a relevant emerging line of research in the field of autism. Bone et al. (2020) show that linguistic markers can be analyzed through AI systems to identify communication patterns associated with ASD. Similarly, Wang et al. (2023) report advances in AI-assisted speech generation systems capable of supporting communication processes in children with autism. Compared with visual or behavioral analysis systems, NLP-based technologies may offer unique opportunities to improve expressive communication and facilitate social interaction, particularly among individuals experiencing language-related difficulties. However, linguistic variability across developmental stages, communication profiles, and cultural contexts represents an important challenge for the generalizability of these systems.

Another relevant observation emerging from this review concerns the methodological robustness and validation procedures of AI models applied to ASD. Although several studies reported promising levels of predictive performance, including high accuracy rates and favorable classification indicators, substantial variability was identified regarding training procedures, validation methods, sample composition, and outcome measures. Importantly, predictive performance should not be interpreted in isolation from methodological rigor. Several studies relied on relatively small or highly specific samples, which may increase the risk of overfitting and reduce the external validity of findings. Models trained on highly homogeneous datasets may perform effectively under controlled research conditions while demonstrating limited applicability in real-world clinical or educational environments.

Moreover, validation procedures varied considerably across studies. While some investigations incorporated independent datasets or cross-validation methods, others relied primarily on internal testing procedures. This variability complicates direct comparison among studies and limits the capacity to determine which approaches demonstrate superior effectiveness under real-world conditions. The lack of methodological standardization across datasets, behavioral indicators, and evaluation metrics further complicates synthesis of findings and reinforces the need for common frameworks capable of improving comparability across future investigations.

In this regard, the methodological quality assessment conducted in the present review revealed that, although most studies demonstrated moderate to high quality, several recurrent limitations persisted. Among the most common concerns identified were restricted sample sizes, limited external validation, dataset imbalance, heterogeneity of performance indicators, and insufficient longitudinal evidence. These findings are particularly relevant because the successful implementation of AI systems in ASD contexts requires not only predictive effectiveness but also stability, replicability, and reliability across different populations and settings.

Beyond methodological robustness, an equally important consideration concerns the practical applicability of artificial intelligence systems in real clinical and educational environments. Across the studies analyzed, artificial intelligence was predominantly conceptualized as a complementary support mechanism rather than a substitute for professional judgment. This distinction is especially relevant in the field of Autism Spectrum Disorder, where diagnosis typically relies on multidisciplinary evaluation processes incorporating developmental history, behavioral observation, standardized instruments, and clinical expertise. Accordingly, the reviewed studies suggest that artificial intelligence systems may serve primarily as screening, classification, and decision-support tools capable of complementing existing professional practices.

Within the domain of early detection and diagnostic support, several studies indicate that machine learning algorithms may facilitate the identification of behavioral indicators associated with autism, particularly in contexts where access to specialized assessment remains limited. Tariq et al. (2019, 2022), for example, demonstrate how digital tools based on machine learning may analyze home videos to identify early behavioral patterns associated with ASD. Similarly, Abbas et al. (2019), Thabtah and Peebles (2020), Rasul et al. (2024), and Jiang et al. (2025) emphasize the growing capacity of predictive models to support diagnostic screening and behavioral classification processes. Nevertheless, although these findings demonstrate considerable technological potential, important caution remains necessary regarding interpretation and implementation. Most of the reviewed studies validated AI systems against already established clinical instruments, including gold-standard procedures such as the Autism Diagnostic Observation Schedule, Second Edition (ADOS-2), and the Autism Diagnostic Interview-Revised (ADI-R). Consequently, artificial intelligence systems should not be interpreted as autonomous diagnostic tools but rather as complementary mechanisms capable of supporting earlier identification, reducing waiting times, and assisting professional decision-making processes.

This distinction becomes especially important when considering the clinical implications of classification errors. False positive classifications may generate unnecessary concern among families and increase pressure on already constrained diagnostic services, potentially leading to inappropriate referrals and unnecessary psychological stress. Conversely, false negative outcomes may delay access to early intervention services during critical developmental periods, thereby limiting opportunities for timely educational and therapeutic support. In this regard, the implementation of AI-based systems in ASD assessment requires careful balancing between predictive efficiency and clinical responsibility. Rather than replacing clinical expertise, artificial intelligence appears most beneficial when functioning within hybrid models that integrate algorithmic prediction with multidisciplinary professional evaluation.

The reviewed literature also suggests substantial educational potential for artificial intelligence systems designed to support learning and social participation among students with ASD. Chen et al. (2021), Hu and Han (2022), and Lan et al. (2024) report promising results regarding adaptive educational environments capable of personalizing learning experiences according to students’ cognitive and behavioral characteristics. Intelligent tutoring systems and AI-supported educational technologies may facilitate individualized instruction, adapt task complexity, provide immediate feedback, and promote social participation within inclusive educational contexts. These characteristics are particularly relevant for students with autism because educational trajectories frequently require differentiated pedagogical responses capable of addressing highly heterogeneous profiles of communication, attention, sensory regulation, and social interaction.

However, despite this promising potential, the reviewed studies also reveal important barriers limiting educational implementation. Several interventions were evaluated within highly controlled research settings, often involving relatively small samples and short intervention periods. Consequently, evidence regarding long-term effectiveness and sustainability in real-world classrooms remains limited. Educational implementation additionally requires adequate technological infrastructure, teacher training, interdisciplinary collaboration, and institutional support, all of which vary considerably across educational systems and socioeconomic contexts. Therefore, although AI-based educational systems may contribute to more personalized learning environments, further validation remains necessary to establish their practical effectiveness within ordinary school settings.

Another important issue emerging from this review concerns equity, bias, and the generalizability of artificial intelligence systems applied to autism. Several authors emphasize that machine learning models are inherently dependent on the quality and representativeness of the datasets used during training procedures. Consequently, biases embedded within datasets may become reproduced or amplified through algorithmic systems. This issue appears particularly relevant in Autism Spectrum Disorder research because ASD populations frequently present substantial demographic, developmental, and cultural heterogeneity.

Most reviewed studies relied on datasets characterized by limited diversity in terms of age, ethnicity, socioeconomic background, and clinical presentation. Additionally, autism research has historically shown a predominance of male participants, despite increasing recognition that girls and women may present distinct behavioral and communicative manifestations often associated with delayed or missed diagnosis. Consequently, AI systems trained primarily on male-dominated datasets may risk underperforming when applied to female populations or individuals presenting less conventional symptom profiles. Similarly, the overrepresentation of clinical or urban populations may reduce applicability in rural or underserved contexts where behavioral characteristics, healthcare access, and environmental conditions may differ substantially.

The challenge of generalizability therefore represents one of the principal limitations of current AI-based autism research. Although predictive models frequently demonstrate promising levels of performance under experimental conditions, their effectiveness may decrease substantially when transferred to heterogeneous real-world environments. Future research should therefore prioritize the development of larger, more diverse, and culturally representative datasets capable of supporting more equitable and generalizable AI systems. Particular attention should also be given to developmental variability across childhood, adolescence, and adulthood, given that behavioral manifestations of autism evolve considerably over time.

Ethical and regulatory concerns also emerge as central issues regarding the integration of artificial intelligence into autism-related contexts. The increasing use of behavioral datasets, home videos, wearable sensors, facial recognition systems, and communication data inevitably raises questions related to privacy, consent, transparency, and accountability. Several reviewed studies relied on audiovisual materials collected within domestic environments, which may involve substantial concerns regarding confidentiality and data security. Families participating in these technologies may not always possess a full understanding of how behavioral information is processed, stored, or reused by algorithmic systems.

Furthermore, concerns surrounding algorithmic transparency remain highly relevant. The limited interpretability of some machine learning and deep learning systems complicates understanding of how predictive outcomes are generated, potentially undermining trust among clinicians, educators, and families. In response to these concerns, explainable artificial intelligence approaches such as those proposed by Jiang et al. (2025) may represent an important direction for future development by improving transparency and facilitating professional interpretation of AI-generated recommendations.

Finally, several limitations of the present review should be acknowledged. First, the heterogeneity of study designs, technological approaches, outcome measures, and validation procedures limited direct comparison among studies and prevented quantitative synthesis through meta-analysis. Second, although a structured methodological quality assessment was conducted using Joanna Briggs Institute critical appraisal tools, variability in methodological quality across included studies may influence interpretation of findings. Third, this systematic review was not prospectively registered in PROSPERO or another international registry. Although the review protocol was developed following PRISMA 2020 methodological recommendations, the absence of prospective registration may limit methodological transparency and should therefore be considered when interpreting the findings.

When positioning the present review within the broader scientific literature, an important distinction should be emphasized. Recent systematic reviews and meta-analyses have primarily focused on specific AI-related domains within Autism Spectrum Disorder, particularly diagnostic prediction models, behavioral monitoring systems, and machine learning approaches based on home-video analysis. In contrast, the present review adopted a broader interdisciplinary perspective by simultaneously examining four major domains of AI application in ASD: early detection and diagnostic support, behavioral and social analysis, educational intervention, and communication support technologies. This broader scope provides a more integrated understanding of how artificial intelligence is progressively being incorporated across clinical, educational, and social dimensions of autism support, thereby extending beyond narrower diagnostic or behavioral frameworks commonly represented in recent reviews.

An important contribution of the present review lies in its broader interdisciplinary scope. Unlike previous reviews primarily centered on diagnostic prediction models or behavioral monitoring systems, the present study integrates evidence across four complementary domains of AI application in Autism Spectrum Disorder: early detection and diagnostic support, behavioral and social analysis, educational intervention, and communication support technologies. This integrative perspective is particularly relevant because individuals with ASD frequently require coordinated responses across clinical, educational, and social environments. Consequently, understanding artificial intelligence exclusively from a diagnostic perspective may offer only a partial understanding of its broader potential. By incorporating educational and communication-related applications alongside diagnostic and behavioral approaches, the present review provides a more comprehensive framework for understanding how AI may contribute to more personalized, adaptive, and inclusive forms of autism support.

Overall, the evidence synthesized in this review suggests that artificial intelligence represents a rapidly expanding and highly promising field for advancing the understanding, detection, educational support, and communication opportunities of individuals with Autism Spectrum Disorder. Nevertheless, the reviewed literature simultaneously demonstrates that technological innovation alone is insufficient to guarantee meaningful implementation. Future progress in this field will depend on the development of methodologically robust, transparent, ethically responsible, and clinically interpretable systems capable of responding to the diversity and complexity of autistic populations. Greater emphasis on external validation, equitable dataset construction, interdisciplinary collaboration, and long-term educational and clinical evaluation will be essential to ensure that artificial intelligence contributes not only to technological advancement but also to genuinely inclusive and evidence-based support for individuals with autism.

## 5 Conclusion

The findings of this systematic review highlight the growing scientific interest in the application of artificial intelligence in the field of Autism Spectrum Disorder (ASD). The studies analyzed indicate that these technologies are primarily being used in four main areas: early detection and diagnostic support, automated analysis of social behavior, the development of adaptive educational tools, and the design of communication support systems. Taken together, these research lines suggest that artificial intelligence may become an important tool for improving assessment, intervention, and support processes for individuals with ASD.

With regard to early detection and diagnostic support, several studies indicate that machine learning algorithms can identify behavioral patterns associated with autism through the automated analysis of data. These tools may facilitate the development of more accessible and efficient screening systems, particularly in contexts where access to specialized clinical assessments is limited. In addition, predictive models based on artificial intelligence may contribute to improving screening processes and supporting diagnostic decision-making among professionals, although they should not replace formal clinical assessment procedures.

Research focusing on the automated analysis of social behavior also demonstrates that artificial intelligence–based technologies can analyze variables related to social interaction, such as eye contact, facial expressions, and visual attention patterns. These tools can complement traditional methods of clinical observation and provide additional information to better understand behavioral characteristics associated with autism.

In the educational field, the results suggest that intelligent educational platforms and adaptive learning systems may contribute to the development of more personalized learning environments for students with ASD. The capacity of these technologies to adjust pedagogical activities to the individual characteristics of each learner may encourage greater participation in educational tasks and support the development of social and communicative skills.

Similarly, advances in natural language processing and assistive communication technologies highlight the potential of artificial intelligence to improve opportunities for social interaction among individuals with autism. Automated analysis of linguistic patterns may support the design of tools that facilitate communication and promote greater inclusion across social and educational contexts.

However, the findings of this review also highlight several limitations that should be considered. These include the methodological heterogeneity among the studies analyzed, the relatively small sample sizes in some investigations, and the need to validate artificial intelligence models in real educational and clinical intervention contexts. Furthermore, the implementation of these technologies raises ethical challenges related to data privacy, algorithm transparency, and the prevention of potential biases in automated systems.

Future research should therefore focus on the development of more robust artificial intelligence models and the use of larger and more representative datasets that may improve the generalizability of findings. In addition, interdisciplinary approaches integrating knowledge from education, psychology, computer science, and health sciences will be essential in order to design technological tools that more effectively respond to the needs of individuals with Autism Spectrum Disorder.

Nevertheless, the findings of this review also indicate that technological progress alone is insufficient to ensure meaningful implementation. Greater methodological robustness, external validation procedures, equitable and representative datasets, and transparent ethical frameworks remain essential to guarantee the responsible integration of artificial intelligence in autism-related contexts. Future developments should prioritize interdisciplinary collaboration between clinical, educational, and technological professionals to maximize both scientific validity and practical applicability.

In conclusion, artificial intelligence represents a significant opportunity to advance the understanding and support of individuals with ASD. However, its implementation should be developed within solid ethical, scientific, and educational frameworks that ensure the responsible use of these technologies and contribute to the development of more inclusive and accessible environments for the autistic community.

## Data Availability

The original contributions presented in the study are included in the article/Supplementary material, further inquiries can be directed to the corresponding author/s.
