# Supplementary material for: Artificial intelligence for autism spectrum disorder: advances in diagnosis, behavior analysis and educational support
Source: Front Neurosci. 2026 Jun 12;20:1832743. doi: 10.3389/fnins.2026.1832743 (PMC13303711; doi:10.3389/fnins.2026.1832743)
Supplement: Supplementary file 1 [file Supplementary_file_1.DOCX]

Supplementary Material

# Supplementary Figures and Tables

**Table 1**

*Complete Search Strategy Across Databases Used in the Systematic Review*

| Database | Date of Search | Complete Search Strategy | Filters Applied | Initial Records Retrieved (n) |
| --- | --- | --- | --- | --- |
| PubMed | May 2025 | ("Autism Spectrum Disorder" OR ASD OR autism) AND ("Artificial Intelligence" OR AI OR "Machine Learning" OR "Deep Learning" OR "Computer Vision" OR "Natural Language Processing") AND (diagnosis OR detection OR education OR intervention OR behavior OR communication) | 2019–2025; full text | 86 |
| Scopus | May 2025 | TITLE-ABS-KEY(("Autism Spectrum Disorder" OR ASD OR autism) AND ("Artificial Intelligence" OR AI OR "Machine Learning" OR "Deep Learning" OR "Computer Vision" OR "Natural Language Processing") AND (diagnosis OR detection OR education OR intervention OR behavior OR communication)) | 2019–2025; articles; full text | 112 |
| Dialnet | May 2025 | "autism and artificial intelligence" OR "autism and machine learning" OR "artificial intelligence and autism diagnosis" OR "AI-based autism education" OR "machine learning and autism detection" OR "artificial intelligence and autism behavior analysis" OR "AI and autism communication support" | 2019–2025; scientific articles; full text | 28 |
| Google Scholar | May 2025 | "autism and artificial intelligence"; "autism and machine learning"; "artificial intelligence and autism diagnosis"; "AI-based autism education"; "machine learning and autism detection"; "artificial intelligence and autism behavior analysis"; "AI and autism communication support" | 2019–2025; English; relevance-based screening; full text when available | 72 |
| Total | 298 | | | |

**Table 2.**

*Quality Assessment and Risk of Bias of Included Studies*

| **Study** | **Main Design / Focus** | **JBI Tool Used** | **Score** | **Quality Level** | **Main Risk of Bias Identified** |
| --- | --- | --- | --- | --- | --- |
| Abbas et al. (2019) | Machine learning / ASD detection | JBI Analytical Cross-Sectional Checklist | 6/8 | Moderate | Limited external validation and possible dataset-specific bias |
| Thabtah (2019) | Behavioral machine learning study | JBI Analytical Cross-Sectional Checklist | 6/8 | Moderate | Heterogeneity of behavioral variables and limited generalizability |
| Tariq et al. (2019) | Home-video ML screening | JBI Diagnostic Accuracy Checklist | 7/9 | High | Potential selection bias and reliance on video quality |
| Voss et al. (2019) | Wearable digital intervention | JBI Randomized Controlled Trial Checklist | 10/13 | Moderate–High | Limited follow-up and implementation constraints |
| Thabtah and Peebles (2020) | Rule-based ML autism detection | JBI Analytical Cross-Sectional Checklist | 6/8 | Moderate | Limited external validation and risk of overfitting |
| Bone et al. (2020) | ML/NLP screening and diagnosis | JBI Diagnostic Accuracy Checklist | 7/9 | High | Variability in linguistic and behavioral measures |
| Washington et al. (2020) | Computer vision / behavioral feature extraction | JBI Diagnostic Accuracy Checklist | 7/9 | High | Limited ecological validation and sample representativeness |
| Chen et al. (2021) | AI-supported educational intervention | JBI Quasi-Experimental Checklist | 7/9 | Moderate–High | Limited sample size and contextual specificity |
| Levy et al. (2021) | Automated facial/emotional analysis | JBI Analytical Cross-Sectional Checklist | 6/8 | Moderate | Limited diversity of samples and possible measurement bias |
| Washington et al. (2021) | Data-driven diagnostics / AI | JBI Diagnostic Accuracy Checklist | 7/9 | High | Generalizability across clinical populations remains limited |
| Alharbi et al. (2022) | ML detection using behavioral datasets | JBI Analytical Cross-Sectional Checklist | 6/8 | Moderate | Dataset imbalance and limited independent validation |
| Tariq et al. (2022) | ML analysis of home videos | JBI Diagnostic Accuracy Checklist | 7/9 | High | Dependence on caregiver-recorded videos and sampling bias |
| Hu and Han (2022) | Intelligent tutoring systems | JBI Quasi-Experimental Checklist | 6/9 | Moderate | Limited real-world educational validation |
| Wang et al. (2023) | AI-supported speech generation | JBI Quasi-Experimental Checklist | 6/9 | Moderate | Small sample and limited longitudinal follow-up |
| Lan et al. (2024) | AI in ASD education | JBI Analytical Cross-Sectional Checklist | 6/8 | Moderate | Heterogeneous educational contexts and limited outcome standardization |
| Rasul et al. (2024) | ML approaches for early diagnosis | JBI Diagnostic Accuracy Checklist | 7/9 | High | Need for broader external validation |
| Jiang et al. (2025) | Explainable AI / multimodal diagnosis | JBI Diagnostic Accuracy Checklist | 8/9 | High | Emerging evidence with limited independent replication |
| Kim et al. (2025) | Deep learning / home-video ASD identification | JBI Diagnostic Accuracy Checklist | 8/9 | High | Potential bias linked to video selection and dataset composition |

**Table 3**
*Description of the included studies.*

| **Author** | **Year** | **Objective** | **Participants** | **Methodology** | **Area of application** | **Results** |
| --- | --- | --- | --- | --- | --- | --- |
| Abbas et al. | 2019 | To develop an AI-based model for identifying behavioral patterns associated with ASD | 97 | Mixed-methods methodology | Diagnostic | The model enabled the classification of behaviors associated with ASD with high accuracy |
| Thabtah | 2019 | To evaluate machine learning algorithms applied to autism diagnosis | 200 | Quantitative methodology | Diagnostic | The algorithms identified behavioral features relevant to the diagnosis of ASD |
| Voss et al. | 2019 | To analyze early behavioral markers using artificial intelligence | 85 | Mixed-methods methodology | Early detection | The system enabled the identification of early indicators of ASD |
| Tariq et al. | 2019 | To design an AI-based digital platform for behavioral assessment | 162 | Mixed-methods methodology | Diagnostic | The tool demonstrated high sensitivity in detecting characteristics of ASD |
| \| Voss et al. \| \| --- \| | 2019 | To analyze the effect of an artificial intelligence-based digital intervention aimed at improving socialization in children with ASD | 71 | Mixed-methods methodology | Communication support | Significant improvement in social interaction and communication skills through the use of intelligent digital devices |
| Washington et al. | 2020 | To use computer vision to analyze patterns of social interaction | 95 | Mixed-methods methodology | Behavioral analysis | The system identified differences in social interaction between children with ASD |
| Thabtah y Peebles | 2020 | To compare different predictive models for the diagnosis of ASD | 110 | Quantitative methodology | Diagnostic | The models demonstrated high accuracy in data classification |
| Bone et al. | 2020 | To analyze linguistic patterns in individuals with ASD using artificial intelligence | 120 | Mixed-methods methodology | Communication | Identification of relevant linguistic differences |
| Levy et al. | 2021 | To analyze facial expressions using artificial intelligence | 65 | Mixed-methods methodology | Behavioral analysis | Automatic identification of emotional patterns |
| Washington et al. | 2021 | To analyze social behaviors using computer vision algorithms | 70 | Qualitative methodology | Social interaction | The system detected differences in visual attention patterns |
| Chen et al. | 2021 | To evaluate an AI-based educational tool designed to improve social skills | 34 | Quantitative methodology | Educational intervention | Improvement in emotional expression and social skills |
| Alharbi et al. | 2022 | To develop a machine learning–based predictive model for diagnosis | 58 | Mixed-methods methodology | Diagnostic | Automated classification of behaviors associated with ASD |
| Tariq et al. | 2022 | To evaluate an AI-based mobile tool for early detection | 312 | Quantitative methodology | Early detection | High accuracy in the identification of early indicators |
| Hu y Han | 2022 | To develop an artificial intelligence–based intelligent tutoring system to support the learning of students with ASD | 45 | Mixed-methods methodology | Educational intervention | The adaptive system enabled the personalization of educational activities and improved the acquisition of social skills |
| Wang et al. | 2023 | To evaluate an artificial intelligence–assisted speech generation system to improve communication in children with ASD | 38 | Mixed-methods methodology | Communication support | Improvement in verbal communication and social interaction through the use of AI-assisted language generation tools |
| Rasul et al. | 2024 | To develop machine learning models for automatic diagnosis | 150 | Quantitative methodology | Diagnostic | Improvement in diagnostic automation |
| Kim et al. | 2025 | A video-based early detection system using deep learning | 120 | Mixed-methods methodology | Early detection | Improved diagnostic accuracy through the use of artificial intelligence |
| Jiang et al. | 2025 | An explainable artificial intelligence framework for autism diagnosis | 100 | Quantitative methodology | Diagnostic | Improved interpretability of artificial intelligence models |

**Table 4**
*Maps of evidences.*

| **Category** | **Reference** | **Year** | **Type of study** | **Key Findings** |
| --- | --- | --- | --- | --- |
| Early detection and diagnostic support | Abbas et al. | 2019 | Observational | Automated identification of behavioral patterns associated with ASD using machine learning algorithms |
|  | Thabtah | 2019 | Experimental | Machine learning models improve autism screening processes |
|  | Alharbi et al. | 2022 | Observational | Improved diagnostic accuracy through the use of machine learning models |
|  | Tariq et al. | 2019 | Experimental | An artificial intelligence–based digital platform for the behavioral assessment of ASD |
|  | Thabtah y Peebles | 2020 | Experimental | Predictive models with high accuracy in the classification of autism-related data |
|  | Rasul et al. | 2024 | Experimental | Improved automation of autism diagnosis through machine learning models |
|  | Jiang et al. | 2025 | Experimental | Development of an explainable artificial intelligence framework to improve the interpretability of diagnostic models for ASD |
|  | Voss et al. | 2019 | Observational | Identification of early behavioral markers associated with ASD |
|  | Tariq et al. | 2022 | Experimental | An artificial intelligence–based mobile application with high sensitivity for early detection |
|  | Kim et al. | 2025 | Experimental | A video-based early detection system using deep learning that improves diagnostic accuracy |
| Behavioral and social analysis | Washington et al. | 2020 | Experimental | Identification of social patterns using computer vision |
|  | Levy et al. | 2021 | Observational | Automated analysis of facial expressions to examine emotional processing |
|  | Washington et al. | 2021 | Observational | Automatic detection of differences in social interaction and visual attention |
| Educational intervention | Chen et al. | 2021 | Experimental | Artificial intelligence–based educational tools promote the development of social skills |
|  | Hu y Han | 2022 | Experimental | An artificial intelligence–based intelligent tutoring system that adapts educational activities to the individual needs of students with ASD, facilitating the acquisition of social skills |
| Communication support | Bone et al. | 2020 | Experimental | Identification of linguistic patterns associated with ASD using artificial intelligence |
|  | Voss et al. | 2019 | Experimental | An artificial intelligence–based digital system that analyzes patterns of social interaction and communication using sensors and wearable devices |
|  | Wang et al. | 2023 | Experimental | An artificial intelligence–assisted speech generation system that improves verbal communication and social interaction in children with ASD |

## Supplementary Figures

**Figure 1**

*Flowchart of study selection.*

**Supplementary Table**

*PRISMA 2020 Checklist*

| Section and Topic | Item | Checklist Item | Location in Manuscript |
| --- | --- | --- | --- |
| Title | 1 | Identify the report as a systematic review | Title / Article type |
| Abstract | 2 | Structured summary including objectives, methods, results, and conclusions | Abstract |
| Introduction | 3 | Describe the rationale for the review | Introduction |
| Introduction | 4 | Provide an explicit statement of objectives | End of Introduction |
| Methods | 5 | Specify inclusion and exclusion criteria | Study Selection Criteria |
| Methods | 6 | Specify information sources/databases searched | Research Strategy |
| Methods | 7 | Present full search strategy | Research Strategy + Supplementary Table 1 |
| Methods | 8 | Specify study selection methods | Research Strategy / Study Selection Criteria |
| Methods | 9 | Describe data collection process | Study Selection Criteria |
| Methods | 10 | Define data items collected | Study Selection Criteria |
| Methods | 11 | Specify risk of bias assessment methods | Quality Assessment and Risk of Bias |
| Methods | 12 | Describe effect measures | Statistical Analysis |
| Methods | 13 | Describe synthesis methods | Statistical Analysis |
| Methods | 14 | Describe reporting bias assessment | Not performed |
| Methods | 15 | Describe certainty assessment | Not performed |
| Results | 16 | Describe study selection process | Figure 1 (PRISMA Flow Diagram) |
| Results | 17 | Cite included studies | Table 1 |
| Results | 18 | Present risk of bias results | Supplementary Table 2 |
| Results | 19 | Present results of individual studies | Results section + Table 1 |
| Results | 20 | Present results of syntheses | Results section + Table 3 |
| Results | 21 | Reporting biases | Not assessed |
| Results | 22 | Certainty of evidence | Not assessed |
| Discussion | 23 | General interpretation of findings | Discussion |
| Discussion | 23a | Limitations of evidence | Discussion |
| Discussion | 23b | Limitations of review process | Discussion |
| Other Information | 24 | Registration and protocol | Materials and Methods |
| Other Information | 25 | Sources of support | Funding |
| Other Information | 26 | Competing interests | Conflict of Interest |
| Other Information | 27 | Availability of data/materials | Supplementary Materials |

**
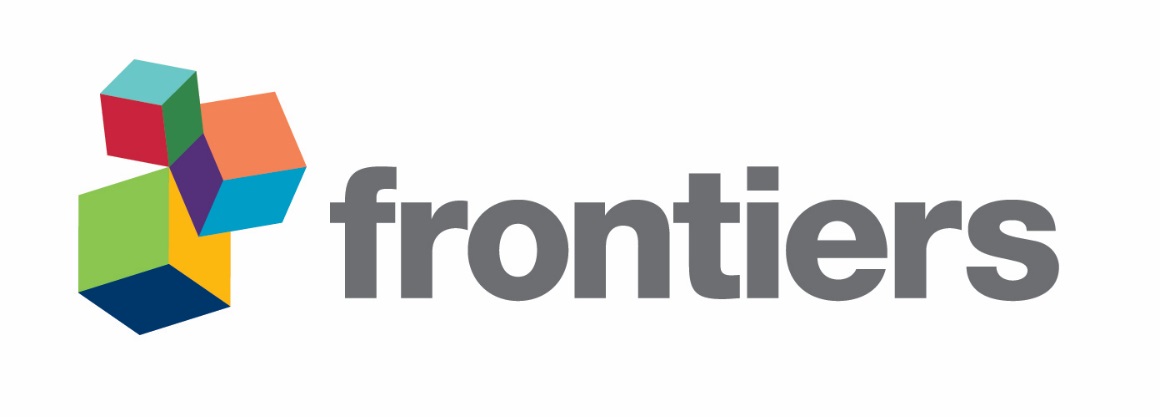
**
